# Stress-Inducible Expression of *HvABF2* Transcription Factor Improves Water Deficit Tolerance in Transgenic Barley Plants

**DOI:** 10.3390/plants13223113

**Published:** 2024-11-05

**Authors:** Rabea Al-Sayaydeh, Jamal Ayad, Wendy Harwood, Ayed M. Al-Abdallat

**Affiliations:** 1 Department of Agriculture Sciences, Faculty of Shoubak College, Al-Balqa Applied University, Al-Salt 19117, Jordan; rabea.sayaydeh@bau.edu.jo; 2 Department of Horticulture and Crop Science, School of Agriculture, The University of Jordan, Amman 11942, Jordan; ayadj@ju.edu.jo; 3 John Innes Centre, Norwich Research Park, Norwich NR4 7UH, UK; wendy.harwood@jic.ac.uk

**Keywords:** *Hordeum vulgare*, inducible expression, promotor, stomatal resistance, transcription factor, water deficit

## Abstract

Barley (*Hordeum vulgare* L.), a major cereal crop grown in arid and semi-arid regions, faces significant yield variability due to drought and heat stresses. In this study, the *HvABF2* gene, encoding an ABA-dependent transcription factor, was cloned using specific primers from water deficit-stressed barley seedlings. Gene expression analysis revealed high *HvABF2* expression in developing caryopses and inflorescences, with significant induction under stress conditions. The *HvABF2* coding sequence was utilized to generate transgenic barley plants with both stress-inducible and constitutive expression, driven by the rice *SNAC1* and maize *Ubiquitin* promoters, respectively. Selected transgenic barley lines, along with control lines, were subjected to water deficit-stress experiments at seedling and flag leaf stages under controlled and greenhouse conditions. The transgenic lines exhibited higher relative water content and stomatal resistance under stress compared to control plants. However, constitutive overexpression of *HvABF2* led to growth retardation under well-watered conditions, resulting in reduced plant height, grain weight, and grain number. In contrast, stress-inducible expression mitigated these effects, demonstrating improved drought tolerance without adverse growth impacts. This study highlights that the stress-inducible expression of *HvABF2*, using the *SNAC1* promoter, effectively improves drought tolerance while avoiding the negative pleiotropic effects observed with constitutive expression.

## 1. Introduction

Barley (*Hordeum vulgare* L.) is one of the most important cereal crops worldwide, serving numerous purposes, including as food, feed, and malting [1]. It is predominantly grown in arid and semi-arid areas and marginal regions characterized by low and variable precipitation, often associated with terminal water deficit and heat stresses [2]. Productivity in these areas varies significantly depending on rainfall amounts and distribution [3]. Accordingly, barley productivity is unstable as it heavily relies on water availability and rainfall distribution throughout the growing season. Furthermore, climate change and its associated conditions, such as high temperatures and low precipitation, are expected to deepen these challenges and negatively impact crop productivity [1]. Future scenarios predict that cereal production, including barley, will be greatly affected by climatic changes, particularly rising temperatures and decreasing precipitation amounts [4].

Limited water supply and high temperatures negatively impact the productivity of many cereals, including barley [5]. The impact of drought stress on plants varies depending on the developmental stage, duration, and severity of the stress and the plant’s ability to adapt to water deficit stress [6,7]. In addition, drought stress affects plant physiological, biochemical, and molecular processes [8,9]. Plants respond to drought stress through alterations in morphology, physiology, and metabolism across different organs [10]. Common features associated with drought stress include growth retardation, impaired membrane integrity, altered pigment content, osmotic adjustment, disrupted water relations, and reduced photosynthetic activity [11,12]. In response to drought, plants accumulate abscisic acid (ABA), a plant hormone associated with abiotic stress tolerance that triggers multiple physiological and biochemical changes, including stomatal closure and the reprogramming of stress-responsive gene expression [13,14].

Numerous stress-responsive genes have been identified in different plant species and were found to be regulated either dependently or independently of ABA [15]. The ABA-dependent regulatory mechanisms are mediated partially by a family of *ABA-responsive element* (*ABRE*)-binding (ABREB) transcription factors, also known as ABA binding factors (ABFs) [16,17]. These transcription factors act as key regulators of ABA signaling and stress-related gene expression, conferring drought tolerance in plants [17,18]. Functional homologs of ABF transcription factors have been studied in various plant species, and their overexpression has been found to improve drought tolerance. For instance, in Arabidopsis, several ABF proteins were shown to regulate numerous stress-responsive genes, and their overexpression in transgenic plants was associated with improved tolerance against various abiotic stresses, including drought, cold, and salinity conditions [19,20]. In rice, OsbZIP46 (OsbZIP46CA1; OsAREB1) was shown to play a pivotal role in ABA action and drought stress tolerance by interacting with other ABF transcription factors and enhancing ABA-responsive gene expression [21]. Additionally, OsbZIP46 was identified as a positive regulator of drought and heat stress responses by upregulating ABA/stress-related genes, although its overexpression negatively affected flowering time in Arabidopsis transgenic lines [22]. Similar findings were reported where transgenic Arabidopsis plants overexpressing *TaAREB3*, encoding an ABF transcription factor from wheat plants, exhibited significant physiological, morphological, and metabolic changes associated with enhanced tolerance to drought and salinity [23]. In barley, several genes encoding ABF transcription factors were identified and reported to play a potential role in the ABA signal transduction pathway and drought stress adaptation [19]. For instance, three barley ABF proteins were found to interact with cellular regulatory proteins (14-3-3) and to play a major role in controlling primary metabolism and ion homeostasis in aleurone layer cells [24]. Therefore, ABF transcription factors are considered powerful targets for the genetic engineering of stress tolerance in plants, as their overexpression can lead to the regulation of a wide array of stress response genes and improved stress tolerance [25].

The overexpression of stress-responsive transcription factors in transgenic plants is associated with pleiotropic effects that significantly affect yield and biomass, limiting their use in crop improvement [26,27]. To overcome these adverse effects, inducible expression using stress-responsive promoters can drive spatial and temporal expression under stress conditions, thus reducing the negative impact of constitutive expression [27,28]. Such inducible expression of stress-responsive transcription factors in different plants has enhanced tolerance to drought, cold, and salinity without negatively impacting growth [29]. For example, the inducible expression of *TaDREB2* and *TaDREB3* driven by the maize *Rab17* promoter in wheat and barley transgenic plants improved water deficit tolerance with fewer pleiotropic effects on plant growth compared to constitutive overexpression genotypes [26]. In another study, the inducible expression of *OsNAC5* significantly enlarged roots and improved water deficit tolerance and grain yield under water deficit conditions [30].

To meet future challenges, developing new varieties that are highly adapted to water deficit conditions is crucial for barley improvement under rainfed conditions and climate change. This can be achieved through the production of new cultivars via conventional breeding strategies or genetic engineering [31]. The application and development of biotechnology have led to new opportunities to enhance the qualitative and quantitative traits of plants [32]. Identifying and utilizing key regulatory genes that confer tolerance to drought are crucial for increasing and stabilizing cereal productivity in dry areas worldwide [25]. Therefore, translating these research advancements into practical applications is essential for the sustainable production of cereal crops in the face of growing environmental challenges. In this study, we analyzed the physiological responses and agronomic performance in selected transgenic barley genotypes engineered to express the *HvABF2* gene, encoding an ABA-responsive transcription factor in barley, under water deficit stress. Transgenic barley plants with inducible or constitutive expression of the *HvABF2*, controlled by stress-responsive or constitutive promoters, were assessed for water deficit tolerance under controlled and greenhouse conditions at two different developmental stages. The results demonstrated that the stress-inducible expression of *HvABF2*-mitigated pleiotropic effects is often associated with constitutive expression and enhanced drought tolerance, making it a promising strategy for improving barley productivity under water-limited conditions.

## 2. Results

### 2.1. Isolation of ABF2 Orthologous in Barley

Using the full-length amino acid sequences of Arabidopsis ABF2 and rice OsbZIP46, an orthologous gene in barley, known as bZIP transcription factor 46-like or ABA-responsive element binding factor 2 (GenBank ID: XP_044960061.1), was identified and subsequently named *HvABF2*. Specific primers were designed to clone the full-length *HvABF2* CDS from stressed barley seedlings, and positive clones were fully sequenced, confirming the encoding of HvABF2. As expected, *HvABF2* encodes a basic leucine zipper (bZIP) domain transcription factor that acts as a positive regulator of ABA signaling and abiotic stress tolerance in plants.

Sequence data analysis of the released barley genome in the Barlex and EnsemblPlants databases indicated that the *HvABF2* gene is located on chromosome 7H and is annotated as *HORVU.MOREX.r3.7HG0663690*. The *HORVU.MOREX.r3.7HG0663690* gene has no splice variants and spans 5070 bp, with a cDNA length of 1395 bp containing four exons and three introns. In terms of genetic variants, 40 alleles were identified in the *HORVU.MOREX.r3.7HG0663690* gene (further information can be found in Gene: HORVU.MOREX.r3.7HG0663690—Variant table—Hordeum_vulgare—Ensembl Genomes 60). Among these, two SNP variants were found to be synonymous variants, while another two SNPs were identified in the first exon as missense mutations (vcZ6O7YG (G/C) and vcZ6O7YH (C/A)), both detected in a *spontaneum_wgs (U)* sample.

**Figure 1:**
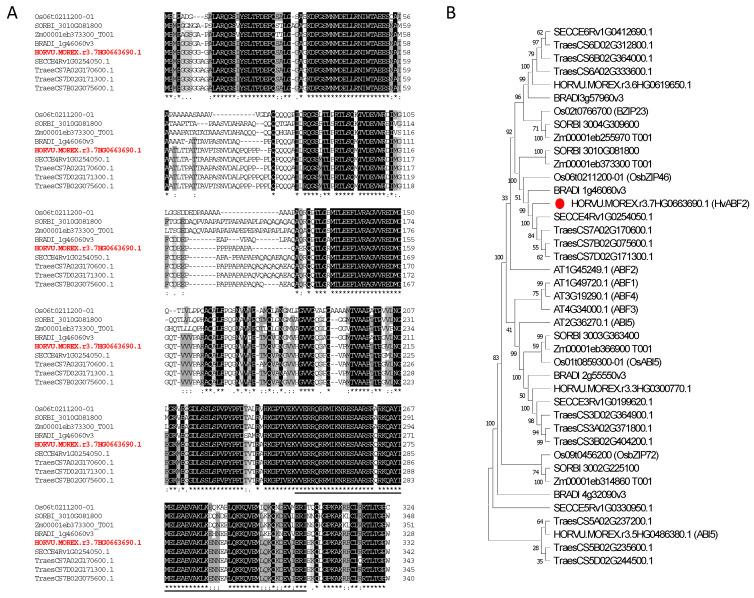
(**A**) Multiple sequence alignment analysis of HvABF2 (HORVU.MOREX.r3.7HG0663690.1) with selected ABF2 orthologous proteins from selected plants (rice: Os06t0211200-01 (OsbZIP46); sorghum: SORBI 3010G081800; maize: Zm00001eb373300; Brachypodium: BRADI 1g46060v3; rye: SECCE4Rv1G0254050.1; wheat: TraesCS7A02G170600.1, TraesCS7B02G075600.1, and TraesCS7D02G171300.1). White letters shaded indicate amino acids that are either 100% identical (black) or identical in at least 80% (dark gray) or identical in at least 60% (light gray), while the underlined letters represent the bZIP domain. (**B**) Phylogenetic analysis of HvABF2 (HORVU.MOREX.r3.7HG0663690.1) with selected ABF2 orthologous and homologous proteins from selected plant species. The position of the HvABF2 protein in the tree is indicated by the red circle.

The deduced complete sequence of HvABF2 contains 332 amino acids and shows the closest identity with TraesCS7B02G075600.1 (94.71%), and it is the closest barley ortholog to OsbZIP46 with 73.49% identity. Analysis of the protein structure of HvABF2 indicated that it belongs to the leucine zipper domain transcription factors superfamily and contains a bZIP domain (located between 248 and 313 in the C-terminal region) involved in DNA binding and protein–protein interaction (Figure 1A).

Multiple sequence alignment of the deduced amino acid sequence of HvABF2 with the closest orthologous protein from selected grasses confirmed the presence of the conserved bZIP domain in the C-terminal parts of the sequences (Figure 1A). Furthermore, phylogenetic analysis placed HvABF2 within the same clade as ABF2 and OsbZIP46, confirming its orthologous relationship with clear clustering with three ABF2 candidates from the wheat plant (Figure 1B).

### 2.2. HvABF2 In Silico Gene Expression Analysis

**Figure 2:**
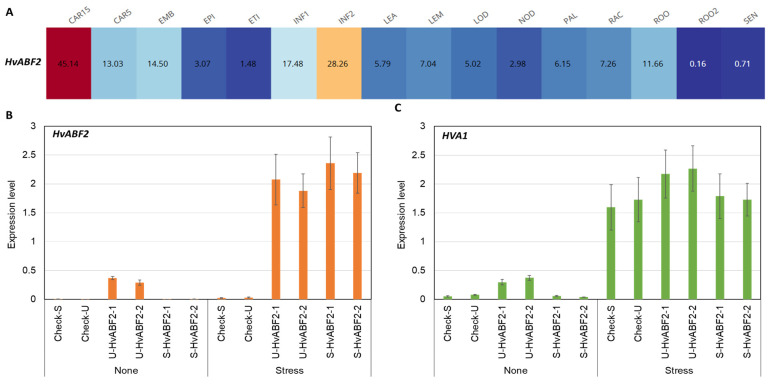
(**A**) In silico expression analysis of *HvABF2* in 16 different tissues using BarleyExpDB (http://barleyexp.com/index.html; accessed on 15 July 2024). Tissue names and corresponding developmental stages are described in Table S1. Expression analysis of (**B**) *HvABF2* and (**C**) *HVA1* genes in different transgenic barley lines in response to water deficit treatment for seven days (Stress) compared to well-watered conditions (None). Transgenic lines include S-HvABF2 (stress-inducible expression of *HvABF2* under *SNAC1* promoter) and U-HvABF2 (constitutive expression of *HvABF2* under *Ubiquitin* promoter), along with corresponding control lines carrying the promoters (Check-S and Check-U) without *HvABF2*. Bars represent SD of two biological samples, each with three technical replicates.

In silico gene expression analysis using RNA-seq data from a 16-tissue experiment in the Morex cultivar revealed high expression levels of *HvABF2* primarily in two plant tissues: developing caryopses (15 days post-anthesis), followed by 10–15 cm long inflorescences from the main tillers of 50-day-old plants (Figure 2A; Table S1). In contrast, the lowest expression levels were detected in root tissue from 10 cm seedlings (Table S1). Interestingly, *HvABF2* expression progressively declined in the radicle and plumule tissue of germinating seedlings. Under drought treatment, several experiments showed a two- to three-fold induction of *HvABF2* expression in leaves of different barley genotypes in response to stress treatments (Table S1). These results suggest that *HvABF2* plays a major role in seed dormancy and the plant’s response to drought stress, highlighting its potential importance in developing stress-tolerant barley varieties.

### 2.3. Stress-Inducible and Constitutive Expression of HvABF2 in Transgenic Barley

To test whether HvABF2 plays a role in drought stress tolerance in barley plants, the coding sequence of *HvABF2* was cloned into a binary plasmid under the control of the *SNAC1* promoter (named S-HvABF2), a stress-inducible promoter from rice, or under the control of the *Ubiquitin* constitutive promoter from maize (named U-HvABF2). Several independent transgenic barley lines carrying a single copy of the transgene were selected for each construct. Additionally, transgenic lines carrying the *SNAC1* and *Ubiquitin* promoters without *HvABF2* were generated and used as controls.

To analyze the inducible and constitutive expression patterns of the *HvABF2* gene in transgenic plants, seedlings were exposed to water deficit conditions for seven days. The expression of the *HvABF2* gene in stressed control plants was induced under water deficit compared to well-watered conditions (Figure 2B), which is in general agreement with the in silico gene expression patterns (Table S1). Under stress conditions, *HVA1*, a stress marker gene in barley, was highly expressed after seven days compared to control plants under normal conditions (Figure 2C). 

For *HvABF2* expression analysis in transgenic plants with stress-inducible or constitutive expression, high expression levels were observed under well-watered conditions in U-HvABF2 transgenic plants compared to control and S-HvABF2 transgenic plants, which showed similar expression values under well-watered conditions (Figure 2C). Under water deficit conditions, the expression of *HvABF2* increased substantially in U-HvABF2 and S-HvABF2 transgenic lines compared to their expression levels under well-watered conditions and in the stressed control plants. These results validate the ability of the *SNAC1* promoter to induce the expression of *HvABF2* in response to stress conditions. For *HVA1*, higher levels were observed in U-HvABF2 transgenic plants under well-watered conditions compared to controls and S-HvABF2, while under stress conditions, high and similar induction levels were observed in all transgenic plants (Figure 2C). 

### 2.4. Stress-Inducible Expression of HvABF2 Improves Drought Tolerance in Barley

To investigate the impact of inducible and constitutive expression of the *HvABF2* gene on the growth and physiological responses of transgenic barley plants under stress conditions, both seedlings and mature plants at the flag leaf stage were subjected to water deficit stress by withholding irrigation for seven days. The stressed transgenic plants were then compared to plants maintained under well-watered conditions. In both experiments, a delay in germination was observed in U-HvABF2 transgenic plants compared to other transgenic lines. Accordingly, U-HvABF2 transgenic plants were sown one week earlier to ensure uniformity in growth.

**Figure 3:**
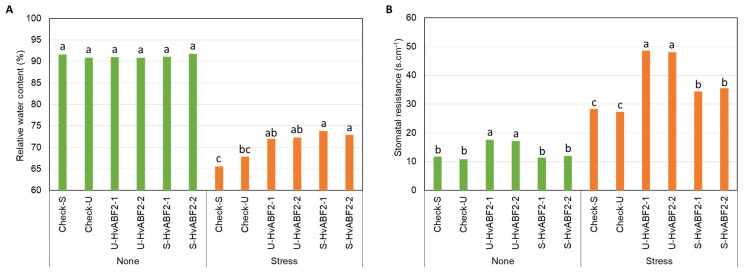
Mean values of relative water content (%) (**A**) and stomatal resistance (s·cm^−1^) (**B**) in different transgenic barley lines at the seedling stage under water deficit treatment (Stress) for seven days, compared to well-watered conditions (None). Transgenic lines include S-HvABF2 (stress-inducible expression of *HvABF2* under *SNAC1* promoter) and U-HvABF2 (constitutive expression of *HvABF2* under *Ubiquitin* promoter), along with corresponding control lines carrying the promoters (Check-S and Check-U) without *HvABF2*. Letters are used to compare means for genotypes within each treatment based on Tukey’s HSD test at *p* ≤ 0.05. The sample size for each genotype within each treatment was four biological replicates (*n* = 4).

At the seedling stage, the control transgenic plants exhibited wilting behavior after seven days of culture, while U-HvABF2 and S-HvABF2 transgenic plants showed better growth under stress conditions. The analysis of variance (ANOVA) for the seedling experiment revealed a significant interactive effect of treatment × genotype for relative water content (RWC) and stomatal resistance (SR) data (*p* ≤ 0.05) (Table S2). Under well-watered conditions, no significant differences were observed in RWC values among the tested plants (Figure 3A). However, the mean values of SR in plants with constitutive overexpression of *HvABF2* (U-HvABF2) showed significantly higher mean values compared to other transgenic lines (Figure 3B). On one hand, S-HvABF2 transgenic plants showed higher mean values for RWC and SR after seven days of stress treatment compared to the control plants. On the other hand, SR mean values of U-HvABF2 lines exceeded those of the control lines approximately by up to 112%. Interestingly, under stress conditions, U-HvABF2 transgenic plants exhibited even higher SR values (24% greater) than the S-HvABF2 plants (Figure 3B).

In the greenhouse experiment, the two-way analysis of variance (ANOVA) revealed a significant interaction between treatment and genotype, affecting both physiological and agronomic traits except for number of tillers (Table S2). The coefficient of variation (CV) values ranged from 1.57% for RWC to 14.28% for grains per spike. For the effect of water treatment on agronomic and physiological traits, the water deficit treatment resulted in lower mean values for all tested agronomic traits compared to well-watered conditions (Table S3). For example, the water deficit treatment significantly reduced plant height mean values from 70.83 cm to 64.54 cm and grain weight from 7.94 g to 0.82 g. For physiological traits, the water deficit treatment resulted in higher mean values for SR and proline content and a lower mean value for RWC compared to well-watered conditions (Table S3).

Regarding the genotype effect, U-HvABF2 transgenic lines showed significantly lower mean values for selected agronomic traits, including plant height, grain number, grain weight, and spike weight, but significantly higher mean values for SR and proline content compared to other tested lines (Table S3). Conversely, S-HvABF2 transgenic lines exhibited higher mean values for plant height, grain number, total plant weight, spike weight, and grain weight compared to other tested lines. For control plants, significantly lower mean values were observed for spike number, RWC, SR, and proline content compared to transgenic lines with either constitutive or stress-inducible *HvABF2* expression (Table S3).

**Figure 4:**
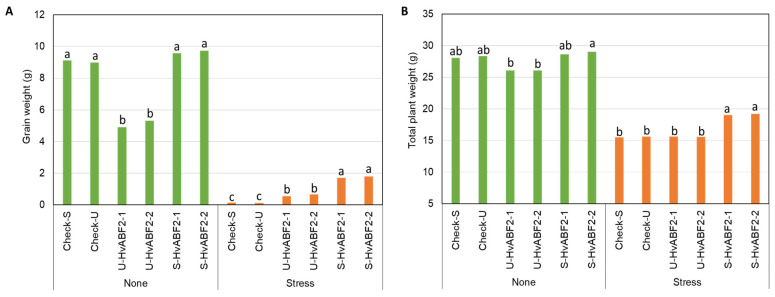
Mean values of grain weight (g) (**A**) and total plant weight (g) (**B**) in different transgenic barley lines subjected to continuous water deficit treatment (Stress) initiated at flag leaf stage compared to well-watered conditions (None). Transgenic lines include S-HvABF2 (stress-inducible expression of *HvABF2* under *SNAC1* promoter) and U-HvABF2 (constitutive expression of *HvABF2* under *Ubiquitin* promoter), along with corresponding control lines carrying the promoters (Check-S and Check-U) without *HvABF2*. Letters are used to compare means for genotypes within each treatment based on Tukey’s HSD test at *p* ≤ 0.05. The sample size for each genotype within each treatment was four biological replicates (*n* = 4).

For agronomic traits, the interaction effect of treatment × genotype revealed significant reductions in grain weight and total plant weight mean values in well-watered U-HvABF2 transgenic plants compared to S-HvABF2 transgenic plants, with reductions ranging from 19% to 66%, respectively (Table S3). As shown in Figure 4A, U-HvABF2 transgenic lines produced significantly lower mean values of grain weight under well-watered conditions when compared to S-HvABF2 and control lines. On the other hand, S-HvABF2 transgenic lines exhibited a significantly higher mean values of grain weight under stress conditions compared to U-HvABF2 lines, which showed higher grain weight values compared to control lines.

A similar pattern was observed for grain number, where U-HvABF2 transgenic plants exhibited lower mean values under well-watered conditions when compared to S-HvABF2 (Table S3). For the number of spikes, control lines produced significantly the lowest mean value under water deficit conditions compared to other tested lines (Table S3). This severe reduction in spike number was associated with lower mean values for grain number in control plants under stress conditions compared to other tested plants. Interestingly, S-HvABF2 transgenic plants exhibited significantly higher mean values for plant height under water deficit stress conditions compared to other transgenic lines, while under well-watered conditions, U-HvABF2 transgenic plants showed the lowest mean value with no significant difference observed between S-HvABF2 and control lines (Table S3). A figure illustrating the effect of water deficit stress on the tested transgenic barley lines is provided in Figure S1.

**Figure 5:**
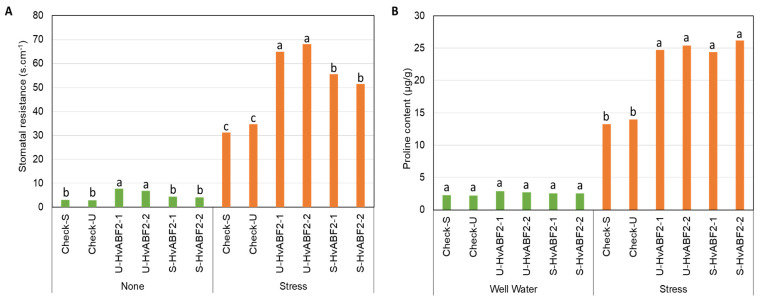
Mean values of stomata resistance (s·cm^−1^) (**A**) and proline content (μg·g^−1^) (**B**) in different transgenic barley lines at the flag leaf stage, measured seven days after the onset of the water deficit (Stress) compared to well-watered conditions (None). Transgenic lines include S-HvABF2 (stress-inducible expression of *HvABF2* under *SNAC1* promoter) and U-HvABF2 (constitutive expression of *HvABF2* under *Ubiquitin* promoter), along with corresponding control lines carrying the promoters (Check-S and Check-U) without *HvABF2*. Letters are used to compare means for genotypes within each treatment based on Tukey’s HSD test at *p* ≤ 0.05. The sample size for each genotype within each treatment was four biological replicates (*n* = 4).

For the interaction effect of treatment × genotype, the comparison of mean values for physiological traits within the same treatment revealed significantly lower mean values under stress conditions for RWC, SR, and proline content in control plants compared to U-HvABF2 and S-HvABF2 transgenic lines (Table S3; Figure 5). Notably, U-HvABF2 exhibited significant differences in SR mean values under normal and stress conditions when compared to S-HvABF2 transgenic lines (Figure 5A), with no significant differences observed for RWC and proline content (Figure 5B). On the other hand, the control plants SR mean values were the lowest compared to other tested transgenic plants, irrespective of stress treatment.

## 3. Discussion

Barley is considered a crop of choice in marginal and rainfed areas, where drought stress is considered a major environmental constraint that limits yield [33]. Understanding the molecular and physiological responses that improve drought tolerance in barley is crucial for breeding strategies aimed at high-yield production in such environments [34]. Recent studies have elucidated the ABA signal transduction pathway that leads to drought tolerance responses and identified several transcription factors involved in ABA signaling [35,36,37]. In this study, the identification and characterization of *HvABF2*, a barley ortholog of *ABF2* and *OsbZIP46*, offer valuable insights into its potential role in stress response mechanisms.

Phylogenetic analysis placed HvABF2 in the same clade as ABF2 and OsbZIP46, indicating close evolutionary relationships and highlighting a potential role in ABA signaling and stress tolerance. Comparative bioinformatics analysis of HvABF2 with ABF2 proteins from other plants revealed a high degree of sequence similarity, particularly in the bZIP domain, suggesting conserved functions in DNA binding and protein interactions, while variations in other regions may confer unique properties that need further investigation [38]. However, understanding HvABF2′s specific function in barley underlines the need for further characterization of its protein structure and its interaction with key regulatory processes. For instance, ABF2 activity in Arabidopsis was found to be associated with ABA-dependent phosphorylation of key amino acids [39]. Biochemical and molecular analyses have confirmed that ABF2 proteins are activated by phosphorylation in an ABA-dependent manner, which is necessary for conferring stress tolerance in Arabidopsis [40]. Therefore, investigating HvABF2 in comparison to its orthologs from other plant species offers insights into its potential role and highlights key areas for future research, especially in understanding how similar ABA-dependent phosphorylation mechanisms might operate in barley to enhance drought tolerance.

The in silico and real-time PCR gene expression analysis of *HvABF2* provided valuable insights into its functional roles and response behavior under stress conditions in barley. High expression levels of *HvABF2* in embryonic tissues suggest its involvement in seed development and dormancy, aligning with previous studies in barley and other species where ABF family members regulate seed maturation and dormancy [24,41,42]. Furthermore, the induction of *HvABF2* under drought conditions supports its role in ABA-dependent stress responses, particularly in enhancing stress tolerance [13,43]. The significant upregulation of *HvABF2* in response to drought stress implies its role in the ABA signaling pathway, orchestrating complex gene networks to confer stress tolerance. Studies in Arabidopsis and rice have shown that the ABA-dependent activation of ABF proteins involves phosphorylation by SnRK2 kinases, which enhances their DNA-binding ability and transcriptional activity [39,40]. Therefore, similar regulatory mechanisms may operate in barley, where HvABF2 may undergo ABA-induced phosphorylation to activate downstream stress-responsive genes and possibly autoregulate its own expression [13,41]. The substantial increase in *HvABF2* expression in transgenic lines with constitutive overexpression confirms the presence of such autoregulatory processes involved in stress-induced expression. Therefore, the expression of *HvABF2* across different tissues and its induction under drought stress underline its role in barley development and stress adaptation.

The genetic transformation of plants is considered a promising technique in crop improvement, particularly for stress tolerance. The induction of *HvABF2* in response to different abiotic stresses indicates the possibility of conferring enhanced tolerance to drought in HvABF2-overexpressing transgenic plants. Testing drought tolerance in transgenic lines indicates that *HvABF2* is stress-inducible and its overexpression enhanced drought tolerance at seedling and reproductive stages under controlled and greenhouse conditions. Similar results were obtained with the overexpression of *HvABF2* orthologs in other plant species, where transgenic lines showed improved growth compared to wild-type plants [35], indicating a conserved role of ABF2 orthologs in drought tolerance across different species. For instance, overexpression of *ABF2* in Arabidopsis was associated with improved drought tolerance and better overall growth under stress conditions [20]. In rice, the overexpression of *OsbZIP46* improved drought tolerance by reducing water loss, thereby improving plant survival and growth under drought conditions [21]. Similarly, transgenic Arabidopsis plants overexpressing *TaAREB3,* a wheat ABF homolog, showed enhanced drought and cold tolerance with increased ABA sensitivity compared to wild-type plants [23]. These results indicate a conserved role of ABF2 orthologs in conferring drought tolerance across diverse plant species by modulating key physiological and molecular responses to drought stress. The conserved nature of these responses highlights the potential of ABF2 orthologs as valuable targets for genetic engineering to enhance drought tolerance in crops.

In this study, the *HvABF2* gene was successfully used to generate stress-tolerant transgenic lines by using two different promoters: the stress-inducible *SNAC1* promoter and the constitutive *Ubiquitin* promoter. The *SNAC1* promoter from rice and its orthologs from various plant species have been previously shown to exhibit stress-inducible expression patterns, mediating cellular mechanisms by specifically inducing gene expression under stress conditions [44,45]. In a recent study, several stress-related *cis*-elements were detected in the promotor region of the *SNAC1* gene, including *ABREs* and *DREs* (dehydration-responsive elements), indicating the involvement of ABA-responsive pathways [46]. In rice plants, the *SNAC1* gene was expressed in response to different abiotic stresses including drought, salinity, and cold, and interestingly, its expression in response to drought was localized predominantly in the stomata [45]. Interestingly, a similar stress-inducible and stomata-specific expression pattern was observed in transgenic barley expressing GFP under the control of the *SNAC1* promoter (Figure S2). This spatial-specific and stress-inducible expression patterns make the *SNAC1* promoter a promising candidate for use in transgenic research, enabling the targeted expression of stress-related genes and potentially minimizing pleiotropic effects commonly associated with constitutive promoters. The constitutive overexpression of stress-responsive transcription factors in transgenic plants can lead to deleterious effects on plant growth and productivity, such as dwarfing, delayed flowering, and reduced grain yield [47]. For instance, the constitutive overexpression of *ANAC055* in Arabidopsis resulted in pleiotropic effects such as delayed flowering and reduced plant height [48]. Similarly, the constitutive overexpression of *OsNAC6* in transgenic rice led to growth retardation and decreased grain yield [49]. In Arabidopsis, the overexpression of two *ABF* genes resulted in improved drought tolerance but also led to stunted growth [50]. These results are in general agreement with our study, where the constitutive overexpression of *HvABF2* in transgenic barley plants resulted in reduced plant height, grain weight, and grain number under normal conditions when compared to control plants (Table S3). These pleiotropic effects associated with *ABF* overexpression under normal conditions were linked to the activation of stress-responsive pathways that divert resources away from growth-related functions [51]. However, the deployment of *SNAC1* stress-inducible promoters surpassed such pleiotropic effects and improved plant growth and yield under normal conditions with a significant effect on tolerance under water deficit conditions. Therefore, the application of stress-inducible promoters for the expression of *HvABF2* proved to be a good strategy to avoid pleiotropic effects under normal conditions compared to constitutive overexpression. Interestingly, while the constitutive expression of *HvABF2* led to reduced plant height and grain weight, the stress-inducible expression did not cause such growth retardation, which is advantageous for avoiding pleiotropic effects often associated with constitutive promoters [15,52,53,54]. These observations align with previous studies where stress-inducible promoters mitigated the adverse growth effects seen with constitutive overexpression of stress-responsive genes [26,30]. For instance, the stress-inducible expression of *TaDREB2* and *TaDREB3* driven by the maize *Rab17* promoter in wheat and barley transgenic plants improved water deficit tolerance with fewer pleiotropic effects on plant growth compared to constitutive overexpression genotypes [26]. In another study, the inducible expression of *OsNAC5* significantly enlarged roots and improved water deficit tolerance and grain yield under water deficit conditions [30,49].

## 4. Materials and Methods

### 4.1. Cloning of HvABF2 in Barley Plant and SNAC1 Promoter in Rice

To identify an *ABF2* orthologous gene in the barley plant, amino acid sequences of the Arabidopsis ABF2 (AREB2: GenBank accession number: NM_179446.5) and the rice AREB2-closest orthologous OsbZIP46 (Os06g0211200: GenBank accession number: XM_015785510.2) protein were used in a TBLASTN search against the NCBI nucleotide sequences database (http://www.ncbi.nlm.nih.gov/BLAST/Blast.cgi; recently accessed on 1 May 2024) with restricting of the search to *Hordeum vulgar* as an organism. Based on the TBLASTN results, the full-length coding sequences (CDS) of the closest *ABF2* orthologous in the barley plant (*HvABF2*: GenBank accession number: XP_044960061.1) were retrieved.

To isolate the full-length CDS of *HvABF2*, specific primers (5′-ATTCCCGGGATGGAGATGCCGGGAGGGA-3′ (*XmaI-HvABF2-Fwd*) and 5′-ATAACTAGTCTACCATGGACCCGTCAGC-3′ (*SpeI-HvABF2-Rev*)) were designed using the NCBI Primer-BLAST tool as described previously [55]. To clone the *HvABF2* CDS, leaf tissues were collected for total RNA extraction from 3-week-old barley cv. Golden Promise seedlings subjected to water deficit by water withholding for 7 days. Total RNA was isolated using an SV Total RNA Isolation System Kit (Promega, Madison, WI, USA) following the manufacturer’s instructions. To synthesize the first-strand cDNA library from the extracted RNA, a SuperScript First-Strand Synthesis System (Invitrogen, Waltham, MA, USA) and *oligo T(18)* primer were used following the manufacturer’s instructions. The full-length *HvABF2* CDS was amplified using PCR in a 25 μL reaction mixture as described previously [55]. The PCR products were visualized in a 1% agarose gel stained with ethidium bromide and extracted from the agarose gel using a Wizard^®^ SV Gel and PCR Clean-Up System (Promega, Madison, WI, USA).

To clone the rice *SNAC1* promoter [45], total genomic DNA was extracted from leaf tissue collected from upland rice line IRAT109 seedlings (kindly provided by Dr. Eklou A. Somado, Africa Rice Center (WARDA)). Specific primers (5′-ATTGGCGCGCCCAACAGTGGAGAGAAAACT-3′ (*AscI-SNAC1p-Fwd*) and 5′- TATGGGCCCCCCCATCGCTTCTTGCTTGC-3′ (*XmaI-SNAC1p-Rev*)) were used to amplify a DNA fragment starting from the base next to the start codon of the *SNAC1* gene to 1373 bp upstream from the start codon using the PCR conditions described above.

The PCR products for *HvABF2* and the *SNAC1* promoter were cloned into a *pGEM^®^-T* Easy Vector System (Promega, Madison, WI, USA) following the manufacturer’s instructions, and then positive recombinants were fully sequenced using the M13 reverse and forward sequencing primers by ABI-3730XL machine by Macrogen (Seoul, Republic of Korea).

### 4.2. Bioinformatics Analysis and Gene Expression Analysis

The *HvABF2* gene structure, chromosomal location, and annotation prediction were analyzed using the EnsemblPlants (https://plants.ensembl.org/index.html; accessed on 15 July 2024) and Barlex databases (https://apex.ipk-gatersleben.de/apex/f%3Fp%3D284:10 accessed on 15 July 2024). For phylogenetic analysis, the deduced amino acid sequence of HvABF2 with barley paralogs and selected orthologs from selected plants were retrieved from the EnsemblPlants database and then aligned using the ClustalW algorithm in MEGA10. A neighbor-joining tree was constructed using parameters described by Alhindi and Al-Abdallat [56].

For gene expression analysis, leaf material from water deficit stressed seedlings, as well as well-watered controls, was collected 7 days after treatment initiation, immediately submerged in liquid nitrogen, and stored at −80 °C until use. The collected tissue was ground into a fine powder using a pestle and mortar in liquid nitrogen. This fine powder was then used to isolate total RNA and for first-strand cDNA synthesis from the extracted RNA as described above. Thereafter, real-time quantitative qRT-PCR was conducted on the cDNA samples using gene-specific primers for *HvABF2* (*ABF2-EXPFwd*: 5′-CTTCGACGAATTCCAGAGCG-3′ and *ABF2-EXPRev*: 5′-GGTTCTTGATCATGCGCCTC-3′). Gene expression was normalized to the *HvActin* gene (*HvActinFwd*: 5′-CTCCTTCACAACCTCAGCTG-3′ and *HvActinRev*: 5′-GGAGCGACGACCTTGATCTT-3′) as a housekeeping reference gene. Real-time PCR reactions were performed in triplicate using a MiniOpticon system (Bio-Rad, Hercules, CA, USA) with a GoTaq Master Mix SYBR Green PCR Kit (Promega, Madison, WI, USA), and each biological treatment was repeated twice. The PCR protocol was as follows: denaturation for 5 min at 94 °C, followed by 42 cycles of denaturation for 45 s at 94 °C, annealing for 30 s at 55 °C, and elongation for 1 min at 72 °C. The relative changes in gene expression were quantified as described previously [57].

Finally, in silico analysis of *HvABF2* gene expression across various growth stages and stress conditions was conducted using RNA-seq data accessed through the Barley Expression Database BarleyExpDB (http://barleyexp.com/index.html accessed on 15 July 2024; [58]).

### 4.3. Plant Material

Golden Promise, a drought- and heat-sensitive barley cultivar [59], was selected to generate transgenic plants due to its high efficiency in callus formation and plant regeneration [60]. To generate transgenic barley lines with stress-inducible and constitutive expression of the *HvABF2* gene, the binary plasmid *pBRACT211* was used. For the constitutive overexpression cassette, the *HvABF2* CDS was excised from the *pGEM^®^-T* Easy using the *XmaI* and *SpeI* enzymes and cloned directly into *pBRACT211* at the *XmaI* and *SpeI* sites downstream of the maize *Ubi* promoter to produce *pBRACT211/Ubi::HvABF2*. For stress-inducible expression, the cloned *SNAC1* promoter was used to replace the *Ubi* promoter in *pBRACT211/Ubi::HvABF2* to produce *pBRACT211/SNAC1::HvABF2* using *AscI* and *SpeI* cloning strategy.

The constructs were then introduced into barley cv. “Golden Promise” using *Agrobacterium*-mediated transformation as described previously [60]. Positive plants carrying a single copy of the transgene were identified as described previously by [61] using quantitative real-time PCR, hygromycin selection, and conventional PCR methods (Figure S2). For this purpose, transgenic seeds from several T1 plants were selected on MS medium containing 50 mg/L hygromycin and lines showing 3:1 segregation for the antibiotic resistance, and *HptII* PCR products (presence or absence) were selected for single-copy analysis using quantitative real-time PCR as described previously [62]. For this purpose, total genomic DNA (gDNA) was extracted from the leaves of each selected transgenic line using a Wizard^®^ Genomic Purification Kit (Promega, Madison, WI, USA) following the manufacturer’s instructions. Thereafter, transgene copy number was determined using RT-qPCR using *HptII* gene-specific primers and *HvActin* gene-specific primers as internal control. Finally, T2 homozygous carrying a single copy of the transgene were identified and further selected and used for the stress experiments.

Transgenic Golden Promise plants harboring the *SNAC1* or *Ubi* promoter and lacking the *HvABF2* gene were generated and used as control. For each construct, two T2 homozygous lines were selected and used in stress experiments. Gene expression levels were analyzed in T2 homozygous seedlings of the selected transgenic lines using the quantitative RT–PCR approach under water deficit and control conditions as described above.

### 4.4. Stress Experiments

Transgenic barley lines with either constitutive or stress-inducible overexpression of the *HvABF2* gene were subjected to water deficit stress conditions and compared to transgenic control lines at two different growth stages. The evaluation was performed through two experiments: one experiment was conducted under controlled growth conditions at the seedling stage and the other in a greenhouse environment at the flag leaf stage. Each experiment was specifically designed to assess the impact of water deficit stress at two different growth stages, providing a comprehensive analysis of *HvABF2*’s role in water deficit stress tolerance.

At the seedling stage (three-leaf-old seedling; Zadoks scale: Z13 [63]), a controlled-growth-conditions experiment was conducted to assess the impact of water deficit stress on the selected transgenic barley genotypes for physiological responses. The experiment took place under controlled growth room conditions with a daily temperature regime of 24 ± 1 °C and a photoperiod of 16/8 (light/dark). For this purpose, two seeds of selected transgenic lines were germinated in 1 L plastic pots filled with acid-washed sand. Both seedlings were maintained at well-watered conditions for one week after germination, and then thinning was performed, leaving one seedling in each pot where the remaining one showed the best vigor and growth. Thereafter, fixed volumes of 1× Hoagland nutrient solution [64] were added to each pot until the end of the experiment. The water deficit stress treatment started when plants reached the third leaf stage (Z13) by water withholding for 7 days. Physiological measurements, including RWC and SR, were analyzed as described in the data collection and statistical analysis section, while gene expression analysis was assessed as described in the bioinformatics and gene expression analysis section.

For the greenhouse experiment, a pot study was conducted to assess the impact of continuous water deficit stress at the flag leaf stage (Zadoks scale: Z37 [63]) on the growth and physiological response of the selected transgenic lines under isolated greenhouse conditions. To establish baseline values for pot moisture management, a gravimetric assessment was performed at the beginning of this study to determine the water-holding capacity of the pots, identifying the moisture level at field capacity according to [65]. This procedure involved saturating the sand in each pot, allowing it to drain, and then measuring the weight of the pots to determine the amount of water retained at field capacity. These measurements provided baseline data, which were used to calculate the precise volumes of water required for irrigation throughout the experiment. Thereafter, two seeds of each transgenic genotype were sown in 10 L plastic pots filled with 8 kg of acid-washed sand. The pots were irrigated weekly with fixed volumes of Hoagland solution until full germination. Thinning was then performed, leaving the most vigorous seedling per pot. During the experiment, pot moisture was managed based on gravimetric data as described previously [66]. Plants were irrigated weekly to maintain pot capacity until the onset of water deficit stress treatment at the flag leaf stage. Thereafter, water deficit stress was applied by withholding water until pot moisture reached 80% depletion of the field capacity, after which pots were rehydrated to field capacity. For stressed plants, this cycle of dehydration and rehydration was repeated whenever pot moisture reached 80% depletion of the field capacity until the end of the experiment. In contrast, non-stressed plants were irrigated whenever soil moisture dropped to 20% depletion from field capacity, and this level was maintained until the end of the experiment. Following this, stressed and non-stressed plants were allowed to grow until they reached physiological maturity (Zadoks scale: GS87 [63]).

### 4.5. Data Collection and Statistical Analysis

Physiological measurements were taken seven days after the onset of the water deficit for both experiments. The RWC was calculated as described previously by Barrs and Weatherley [67]. For this purpose, a fully expanded leaf was sampled from each treated plant (4 replicates per treatment) after 7 days of water deficit stress initiation for both greenhouse and controlled experiments. Leaf fresh weight was immediately recorded after leaf excision, and the excised leaves were then soaked in distilled water for 6 h at room temperature under darkness. Thereafter, the leaves were dried on dry paper towel to remove water, and the turgid weight was recorded. Leaf dry weight was finally recorded after drying leaf samples for 24 h at 70 °C. The leaf relative water content was calculated according to the formula of Barrs and Weatherley (1962) [67]. Stomatal resistance (SR) in treated plants (4 replicates per treatment) was measured after 7 days of the initiation of water deficit stress for both greenhouse and controlled experiments by using a “steady-state” porometer (AP4 model, Delta T devices) attached to the abaxial side of leaves. The readings were taken at midday on three fully expanded leaves per treatment situated at different positions on the canopy. For proline concentration, a fully expanded leaf (1.0 g) was collected at 7 days post-initiation of the water deficit stress for the greenhouse experiment (4 replicates per treatment) and used to determine proline levels following Bates et al. (1973) [68].

For the greenhouse experiments, treated genotypes were harvested at the physiological maturity stage, and the following agronomical parameters were collected: Plant height (PH in cm) was measured from the plant base to the top of the inflorescence, excluding awns. Spike length (SL in cm) was measured from the spike base to the top of it, excluding awns. Total plant weight (TPW in g) was recorded as the total weight of the whole above-ground material, including grains harvested from each pot. Spike weight (SW in g) was measured as the weight of harvested spikes from each plant in the pot. Grain weight (GW in g) was measured as the weight of harvested grains after threshing from each plant in the pot. Grain number (GN) was determined as the total number of grains counted from all harvested spikes from each plant in the pot. Spike number (SN) was the final number of spikes counted from each plant in the pot. Tiller number (TN) was the final number of tillers counted from each plant in the pot.

For both experiments, the treatments included the selected transgenic events and two water levels (well-watered and water deficit stress), and the treatments were arranged in a factorial experiment within a completely randomized design (RCBD) with four biological replications per treatment. Physiological and agronomical data were analyzed using GenStat software (Release 16.1, 2013; VSN International, Ltd., Oxford, UK). A two-way analysis of variance (ANOVA) was performed to assess the interaction effects between water treatments and genotypes. The statistical model included water treatment, genotype, and their interaction as fixed effects, with block treated as a random effect. The Tukey HSD test (*p* ≤ 0.05) was used for mean separation.

## 5. Conclusions

In this study, the *HvABF2* gene, an ABA-dependent transcription factor, was successfully isolated and characterized in barley. The gene exhibited high expression in developing caryopses and embryos, with a significant induction under water deficit conditions. Transgenic barley lines expressing *HvABF2* either constitutively or under the control of a stress-inducible promoter (*SNAC1*) showed enhanced drought tolerance at two different growth stages. Specifically, transgenic barley plants with constitutive overexpression of *HvABF2* exhibited delayed germination and pronounced reductions in agronomic traits such as plant height, grain weight, and total plant weight, suggesting a trade-off between stress tolerance and growth. Conversely, transgenic barley lines with stress-inducible expression of *HvABF2* displayed better agronomic performance under stress, indicating that stress-inducible expression of *HvABF2* may provide a more balanced approach to managing stress tolerance, with less impact on growth and yield. This study highlights the potential of using stress-inducible promoters to improve drought tolerance in barley, providing a viable strategy to mitigate the negative pleiotropic effects often associated with constitutive gene expression. The approach of the stress-inducible expression of *HvABF2* offers a promising pathway for developing stress-tolerant cereal varieties for arid and semi-arid areas. Further research could explore the application of this strategy in other cereal crops, paving the way for broader agricultural resilience to abiotic stresses.

## Data Availability

The datasets supporting the results of this article are included in the manuscript and Supplementary Materials, and further enquiries can be requested from Ayed M. Al-Abdallat.

## Supplementary Materials

The following supporting information can be downloaded at: https://www.mdpi.com/article/10.3390/plants13223113/s1, Table S1: In silico gene expression analysis of *HvABF2* using the BarleyExpDB database; Table S2: Mean squares from the combined analysis of variance for the effect of water deficit treatment on various transgenic barley lines; Table S3: Mean values of eight agronomic and three physiological traits from the greenhouse experiment; Figure S1: Growth performance of different transgenic barley lines subjected to water deficit treatment at flag leaf stage for seven days, compared to well-watered conditions. Transgenic lines include (A) control; (B) U-HvABF2-1; (C) U-HvABF2-2; (D) S-HvABF2-1; (E) S-HvABF2-2. S-HvABF2: stress-inducible expression of *HvABF2* under *SNAC1* promoter; U-HvABF2: constitutive expression of *HvABF2* under *Ubiquitin* promoter; corresponding control lines carrying the promoters without *HvABF2*; Figure S2: Microscopy analysis of GFP expression in stomata of transgenic barley plants expressing GFP under the control of the SNAC1 promoter after 1 h of water deficit stress; Figure S3: Example of screening T1 and T2 transgenic plants using antibiotic resistance, conventional PCR, and real-time PCR assays: (A) Agarose gels displaying the results of *HptII* presence/absence in U-HvABF2#T1-06 T1 plants, which is segregated in a 3:1 ratio for antibiotic resistance and PCR assays; the results for three selected T2 lines (null, homozygous, and heterozygous) derived from U-HvABF2#T1-06 are shown. (B) Copy number validation was conducted using the real-time PCR method, following the protocol outlined by Weng et al. (2004) [62].
