# Supplementary material for: Stress-Inducible Expression of HvABF2 Transcription Factor Improves Water Deficit Tolerance in Transgenic Barley Plants
Source: Plants (Basel). 2024 Nov 5;13(22):3113. doi: 10.3390/plants13223113 (PMC11597383; doi:10.3390/plants13223113)
Supplement: Supplementary file 1 [file plants-13-03113-s001.zip › Suppl. Figures.pdf]

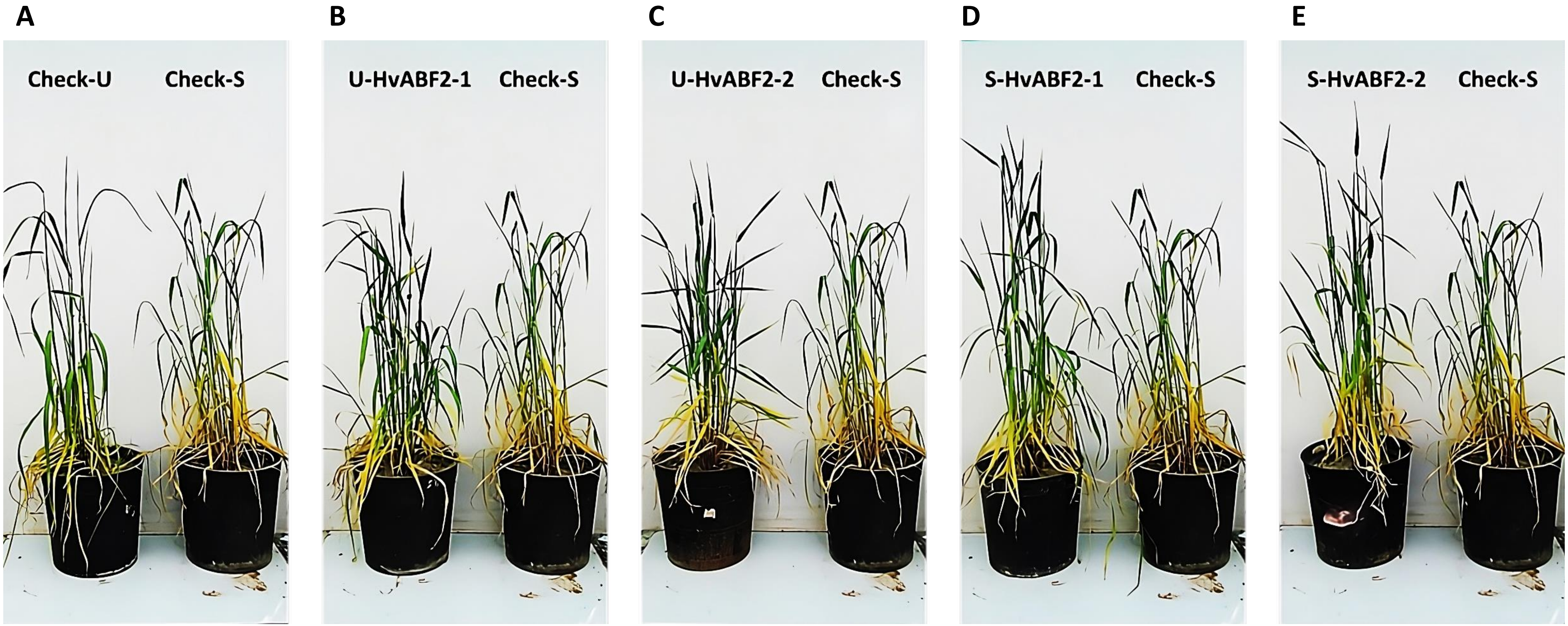

Figure S1. Phenotypes of different transgenic barley lines under water deficit treatment and well-watered conditions at the flag leaf stage. The images were captured seven days after the initiation of water deficit treatment. Transgenic lines include: (A) Checks; (B) U-HvABF2-1; (C) U-HvABF2-2; (D) S-HvABF2-1; (E) S-HvABF2-2. S-HvABF2: stress-inducible expression of *HvABF2* under *SNAC1* promoter; U-HvABF2: constitutive expression of *HvABF2* under *Ubiquitin* promoter; Corresponding check lines carrying the promoters without *HvABF2*.

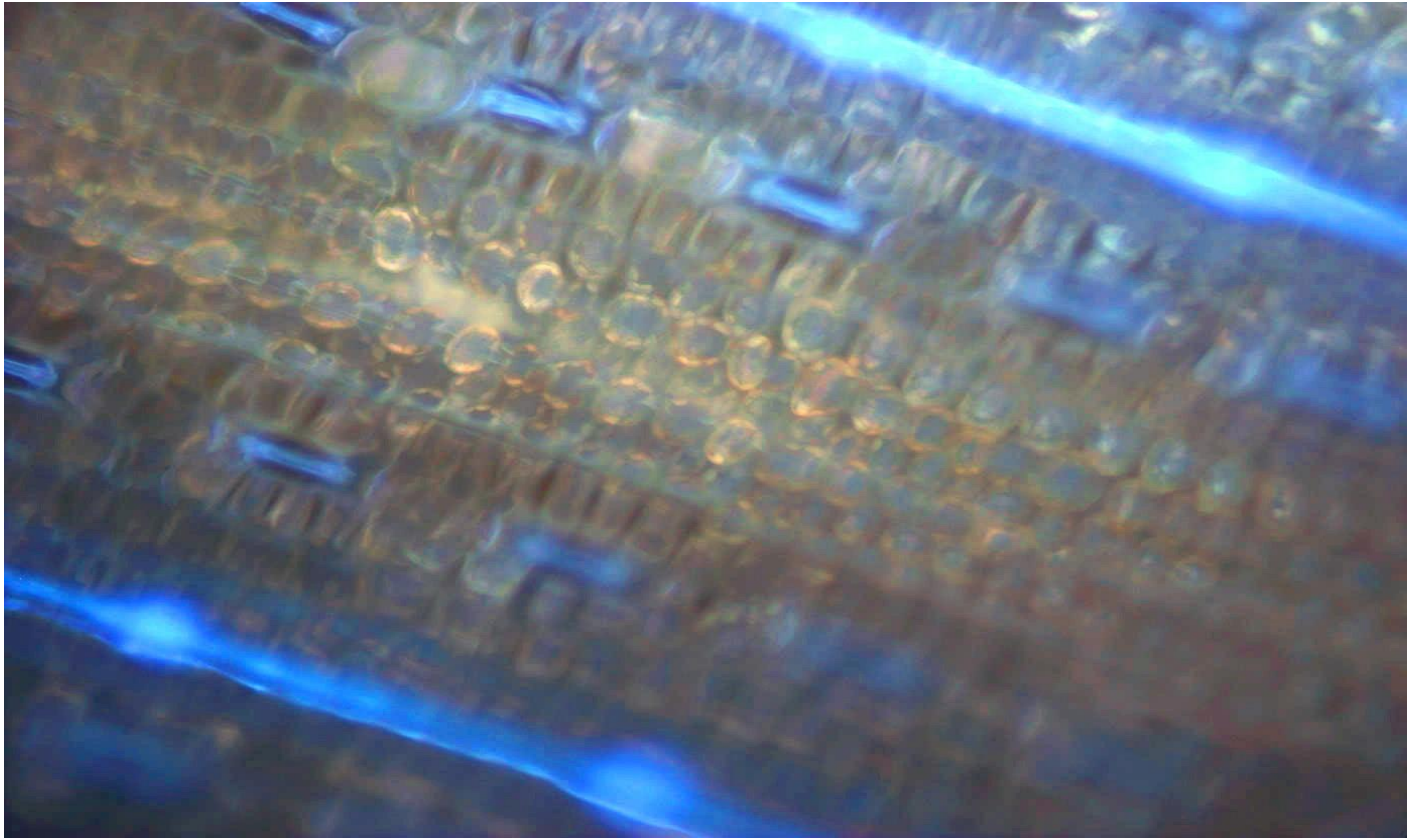

Figure S2. Microscopy analysis of GFP expression in stomata of transgenic barley plants expressing GFP under the control of the SNAC1 promoter after 1 hour of water deficit stress.

A)

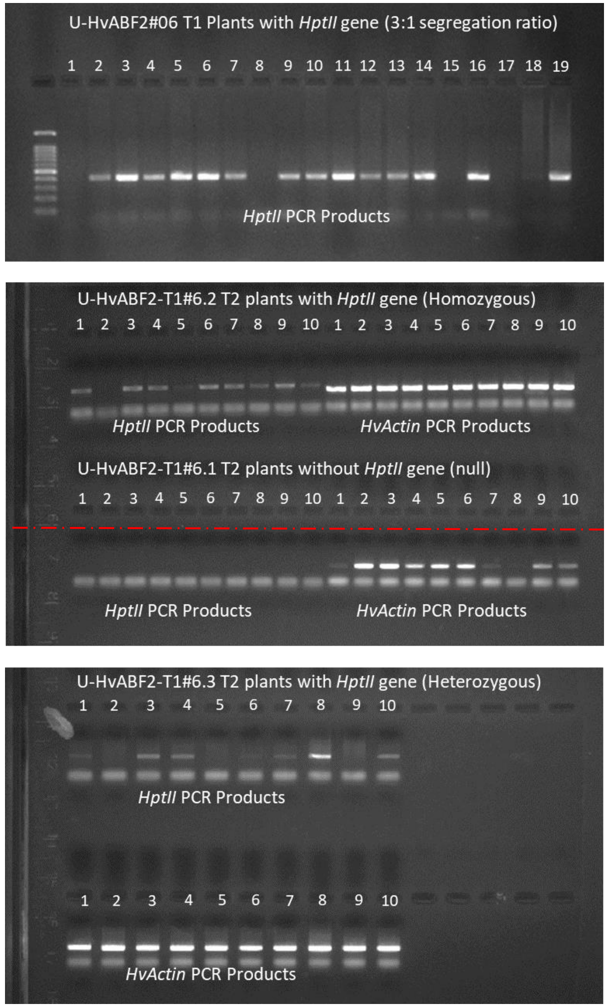

B)

ID	Sample	CTX	IX	SX	CTR	IR	SR	X0/R0	Copies Hptii
U-HvABF2-T1#6.01	A01	N/A	27.664	-3.392	27.91	27.926	-3.380	#VALUE!	#VALUE!
U-HvABF2-T1#6.02	A02	25.51	27.664	-3.392	25.65	27.926	-3.380	0.92	2
U-HvABF2-T1#6.03	A03	26.57	27.664	-3.392	25.43	27.926	-3.380	0.38	1
U-HvABF2-T1#6.04	A04	26.99	27.664	-3.392	26.14	27.926	-3.380	0.47	1
U-HvABF2-T1#6.05	A05	26.58	27.664	-3.392	25.58	27.926	-3.380	0.42	1
U-HvABF2-T1#6.06	A06	26.83	27.664	-3.392	26.21	27.926	-3.380	0.55	1
U-HvABF2-T1#6.07	A07	25.44	27.664	-3.392	25.44	27.926	-3.380	0.83	2
U-HvABF2-T1#6.08	A08	N/A	27.664	-3.392	26.88	27.926	-3.380	#VALUE!	#VALUE!
U-HvABF2-T1#6.09	A09	26.36	27.664	-3.392	25.42	27.926	-3.380	0.44	1
U-HvABF2-T1#6.10	A10	26.26	27.664	-3.392	26.34	27.926	-3.380	0.88	2
U-HvABF2-T1#6.11	B01	26.07	27.664	-3.392	26.03	27.926	-3.380	0.81	2
U-HvABF2-T1#6.12	B02	27.16	27.664	-3.392	26.07	27.926	-3.380	0.40	1
U-HvABF2-T1#6.13	B03	27.61	27.664	-3.392	26.78	27.926	-3.380	0.48	1
U-HvABF2-T1#6.14	B04	26.94	27.664	-3.392	26.61	27.926	-3.380	0.67	1
U-HvABF2-T1#6.15	B05	N/A	27.664	-3.392	27.86	27.926	-3.380	#VALUE!	#VALUE!
U-HvABF2-T1#6.16	B06	26.31	27.664	-3.392	25.36	27.926	-3.380	0.44	1
U-HvABF2-T1#6.17	B07	N/A	27.664	-3.392	27.33	27.926	-3.380	#VALUE!	#VALUE!
U-HvABF2-T1#6.18	B08	27.21	27.664	-3.392	27.39	27.926	-3.380	0.94	2
U-HvABF2-T1#6.19	B09	26.76	27.664	-3.392	25.73	27.926	-3.380	0.41	1
GP-WT	D01	N/A	27.664	-3.392	26.49	27.926	-3.380	#VALUE!	#VALUE!
GP-WT	D02	N/A	27.664	-3.392	26.15	27.926	-3.380	#VALUE!	#VALUE!

Figure S3. Example of screening T1 and T2 transgenic plants using antibiotic resistance, conventional PCR, and Real-Time PCR assays: A) Agarose gels displaying the results of HptII presence/absence in U-HvABF2#T1-06 T1 plants, which segregated in a 3:1 ratio for antibiotic resistance and PCR assays; The results for three selected T2 lines (null, homozygous, and heterozygous) derived from U-HvABF2#T1-06 are shown. B) Copy number validation was conducted using the Real-Time PCR method, following the protocol outlined by Weng et al., 2004.
